# Relationships between disease severity and measures of health status in people with interstitial lung disease in India: an observational study

**DOI:** 10.1038/s41598-025-01877-4

**Published:** 2025-05-16

**Authors:** Revati Amin, K. Vaishali, G. Arun Maiya, Aswini Kumar Mohapatra, Vishak Acharya, Marita T. Dale, Jennifer A. Alison

**Affiliations:** 1 Department of Physiotherapy, Kasturba Medical College Mangalore, Manipal Academy of Higher Education, Manipal, India; 2 Department of Physiotherapy, Manipal College of Health Professions, Manipal Academy of Higher Education, Manipal, India; 3 Department of Respiratory Medicine, Kasturba Medical College Manipal, Manipal Academy of Higher Education, Manipal, India; 4 Department of Pulmonary Medicine, Kasturba Medical College Mangalore, Manipal Academy of Higher Education, Manipal, India; 5 Sydney School of Health Sciences, Faculty of Medicine and Health, The University of Sydney, Sydney, Australia; 6 Allied Health, Sydney Local Health District, Sydney, Australia

**Keywords:** Respiratory tract diseases, Medical research

## Abstract

The severity and progression of Interstitial Lung Disease (ILD) can vary due to environmental, cultural and genetic factors. The relationship between disease severity and factors that determine the health status of people with ILD living in lower middle-income countries like India has not been evaluated. This study aimed to determine whether there were relationships between disease severity with functional exercise capacity and Health Related Quality of Life (HRQoL) among people with ILD in India. This was a prospective, single center observational study. All participants performed Pulmonary Function Test (PFT) including Forced Vital Capacity (FVC) % predicted, Diffusing Capacity of the Lung for Carbon Monoxide (D_L_CO) % predicted, 6 min Walk Distance (6MWD), St. George’s Respiratory Questionnaire (SGRQ), modified Medical Research Council (mMRC) dyspnoea scale. Eighty participants with ILD were recruited from September 2020 and December 2022. There were strong correlations between 6MWD with D_L_CO % pred (Spearman rho 0.891) and between SGRQ Total score and D_L_CO % pred (Spearman rho 0.906). There were no correlations between 6MWD and FVC % pred (Spearman rho 0.206) or between SGRQ and FVC % pred (Spearman rho 0.113). This study demonstrated strong correlations between disease severity measured by D_L_CO % pred with both functional exercise capacity and HRQoL in people with ILD in India.

## Introduction

Interstitial lung disease (ILD) is an umbrella term for a large group of diseases that cause interstitial fibrosis of the lungs^1^ ILDs are characterized by symptoms such as dyspnea, fatigue, and reduced exercise tolerance, which can greatly impact patients’ quality of life^2–4^ The progression of ILDs can vary, from being stable, to slowly or rapidly progressive, with the overall prognosis being poor^5,6^ Despite recent improvements in treatment options and diagnostic techniques, the mortality rate for ILDs globally remains high^7^.

Clinical evaluation, histopathologic sampling, and high-resolution computed tomography (HRCT) are effective methods for detecting and classifying ILDs. Pulmonary function testing (PFT), particularly the diffusing capacity for carbon monoxide (D_L_CO), is the most sensitive method for assessing the progression of ILDs^8^ D_L_CO is regarded as the most sensitive approach for measuring ILD progression because it precisely evaluates gas exchange efficiency, revealing early anomalies even before significant changes in lung capacity or airflow become apparent^8^ D_L_CO, as compared to FVC, is directly associated with the severity of the disease, especially in diseases like IPF, where decreases in D_L_CO are strongly correlated with a worse prognosis, higher rates of morbidity, and mortality. Additionally, it provides early impairment detection since it is more sensitive to the microvascular alterations linked to fibrosis. D_L_CO is an important tool in clinical practice and research because it is a critical prognostic indicator that aids in tracking the evolution of the disease and is linked to a decrease in exercise capacity, an increase in symptoms, and a decreased survival rate^8–14^ Strong correlations have been found between decreasing values of D_L_CO, disease severity and increased morbidity in ILDs, particularly in cases of idiopathic pulmonary fibrosis (IPF)^9^ Forced vital capacity (FVC) is another standard measure of pulmonary function for evaluating ILDs with a reduction in FVC over time considered a reliable indicator of disease progression^15^ and an independent predictor of mortality in patients with IPF^15,16^.

The 6-minute walk test (6MWT) is used to measure functional exercise capacity in people with ILD and is known to be a predictor of mortality^17^ Reduced lung volumes caused by irreversible pathological changes in the lungs can lead to the debilitating symptoms of dyspnea and fatigue and reductions in exercise capacity, muscle strength, and endurance, which can negatively impact patients’ physical and mental well-being and quality of life^18–21^ Studies have shown a relationship between disease severity and functional exercise capacity, which helps to understand the effects of the disease on patients,^22–25^ but there are no studies in an Indian population.

In developing countries like India, ILDs are often underdiagnosed at early stages due to tests and investigations like D_L_CO and HRCT not typically being performed due to under resourcing, lack of testing unless, lack of standardized approach for diagnosis and a tendency to rely on a single physician’s judgment. Additionally, factors such as patient reluctance, hesitancy, and the assumption of alternative diagnoses further contribute to delays in accurate diagnosis and management^26–29^ Using other more readily accessible tests that could indicate the ILD severity may be of benefit. The relationship between disease severity and HRQoL may be impacted by these diagnostic gaps because severity may be underestimated or missed in its early stages, resulting in distinct patient experiences when compared to higher-income countries^26–29^ ILDs in India are largely caused by environmental exposures, including smoke from biomass fuels, outdoor air pollution, and occupational exposures. These exposures may have a distinct impact on the course and symptoms of disease and are different from those frequently studied in high-income countries. Patient perceptions and reports of their quality of life may also be influenced by cultural variables, such as expectations for caregiving, familial obligations, and the stigma attached to chronic illnesses. Furthermore, the severity and progression of ILDs can vary due to environmental, cultural and genetic factors^26–29^.

Currently, the relationships between disease severity and factors that determine the health status of people with ILD living in lower-to middle-income countries like India have not been evaluated. Understanding these relationships may provide a measure of disease severity when other tests and investigations are unavailable. The aim of the study was to determine the relationship between disease severity, functional exercise capacity and health-related quality of life (HRQoL) in people with ILD in India, and to determine whether there were differences in these relationships according to sex and time since diagnosis.

## Methods

### Participants

People diagnosed with ILD who attended Kasturba Hospital, Manipal were recruited between September 2020 and December 2022. To be included in the study participants had to have an ILD diagnosed by HRCT,^30,31^ be able to perform a 6MWT, not had an acute exacerbation of ILD or any infection within the last 4 weeks and not have heart failure. The study was approved by the Kasturba Medical College and Kasturba Hospital Institutional Ethics Committee (IEC: 320/2020) and was registered in the Clinical Trials Registry of India (CTRI/2020/09/027788).^32^ Written informed consent was obtained from all the study participants and study was conducted in accordance with Declaration of Helsinki. In this study, ILD subtypes have been determined employing a multidisciplinary diagnostic (MDD) approach that involves collaboration among pulmonologists, radiologists, rheumatologists, and pathologists as needed. Clinical history, physical examination, serological testing, high-resolution computed tomography (HRCT) results, and, in certain situations, histological confirmation were used to classify ILDs. Because of its specific pathophysiology and treatment implications, RA-ILD was examined as a separate subgroup to highlight variations in clinical features and progression when compared to other forms of CTD-ILD.

The study classified patients with Non Specific Interstitial Pneumonia (NSIP) according to whether the illness was idiopathic or because of an underlying CTD. Patients with idiopathic NSIP were categorized separately, whereas those with secondary NSIP were included in the CTD-ILD category. This distinction was developed in order to enable the investigation of disease patterns and guarantee accurate grouping based on etiology.

A sample of 80 patients was chosen based on specified inclusion and exclusion criteria associated with the diagnosis of ILD.

This study is a subset of a larger study which is a randomized controlled trial (RCT)^32^ We calculated sample size for the RCT, the details have been published^32^ We recognize that the results generalizability may be constrained by the comparatively small sample size in terms of statistical power. Prior to the investigation, we did, however, perform a power calculation to make sure the sample size was adequate to identify clinically significant variations in key findings.

On the same day, all participants completed Pulmonary Function Tests (PFTs) consisting of spirometry (FEV_1_ and FVC), static lung volumes and D_L_CO which were compared to predicted normal values,^33,34^ a 6MWT according to standard protocol, with two tests conducted 30 min apart and the best distance used in analyses,^35^ the mMRC dyspnoea scale^36^ and the St George’s Respiratory Questionnaire (SGRQ)^33,37^.

PFTs, including spirometry and D_L_CO measurements, were performed using the Koko Spirometer (USA). This equipment was calibrated daily according to the manufacturer’s guidelines to ensure accuracy and consistency throughout the study. The predicted values for pulmonary function parameters were calculated using the ERS/ATS reference equations, which were selected to align with current clinical guidelines and ensure standardization across our measurements. Furthermore, to eliminate inter-device variability, all patients were assessed using the same spirometer. The tests were conducted by trained technicians who strictly adhered to the ATS/ERS guidelines for PFT performance^33,34^ This included proper patient preparation, adherence to technical standards, and the performance of at least three acceptable maneuvers for both spirometry and D_L_CO measurements. Only tests meeting the ATS/ERS quality criteria were included in the analysis. For spirometry, this required at least three reproducible maneuvers with a difference of ≤ 150 mL between the two highest FEV1 and FVC values. For D_L_CO, measurements were accepted if they met the criteria of a breath-hold time of 8–12 s, a washout volume of ≥ 0.75 L, and a sample collection time of 2–4 s. The mMRC is a self-assessment of the level of dyspnoea during mobility on a 0–4 scale with 0 rating ‘breathless while performing strenuous activities’ to 4 rating ‘too breathless to leave the house’ or ‘breathless when dressing’. The SGRQ examines the impact of respiratory disease across three domains: symptoms, impact and activity. The total score directly corresponds to the level of disability and has been validated in people with ILD, with a higher score indicating worse HRQoL^37^.

Given that the development of symptoms and the course of the disease in ILD usually intensify after the first few years of diagnosis, the 3-year diagnosis cut-off was chosen. This period of time permits enough disease progression for individuals to have had quantifiable changes in lung function, functional capability, and quality of life. Furthermore, the 3-year mark is a suitable window for studying the relationship between severity of the disease and outcomes because it is during this time that clinicians usually observe the early phases of disease stability or advancement.

### Data analysis

Data analysis was performed using Jamovi 2.2.5 software. The Shapiro-Wilk test was used to determine normality of the variables. Based on normality assessment, mean, median, standard deviation (SD), interquartile range (IQR) were used to describe the findings. Spearman’s signed rank test was used to compute the correlation between 6MWD and D_L_CO % predicted and 6MWD and FVC % predicted. Chi square test was used to establish association between variables. Receiver Operating Characteristics (ROC) curve, specificity and sensitivity tests were used to test accuracy. We determined the strength of correlation on absolute values of r with a correlation defined as strong when *r* = 0.6 and above, moderate when *r* = 0.40–0.59 and weak when *r* = 0.2–0.39^38^ Statistical significance was defined as *p* < 0.05.

## Results

**Fig. 1:**
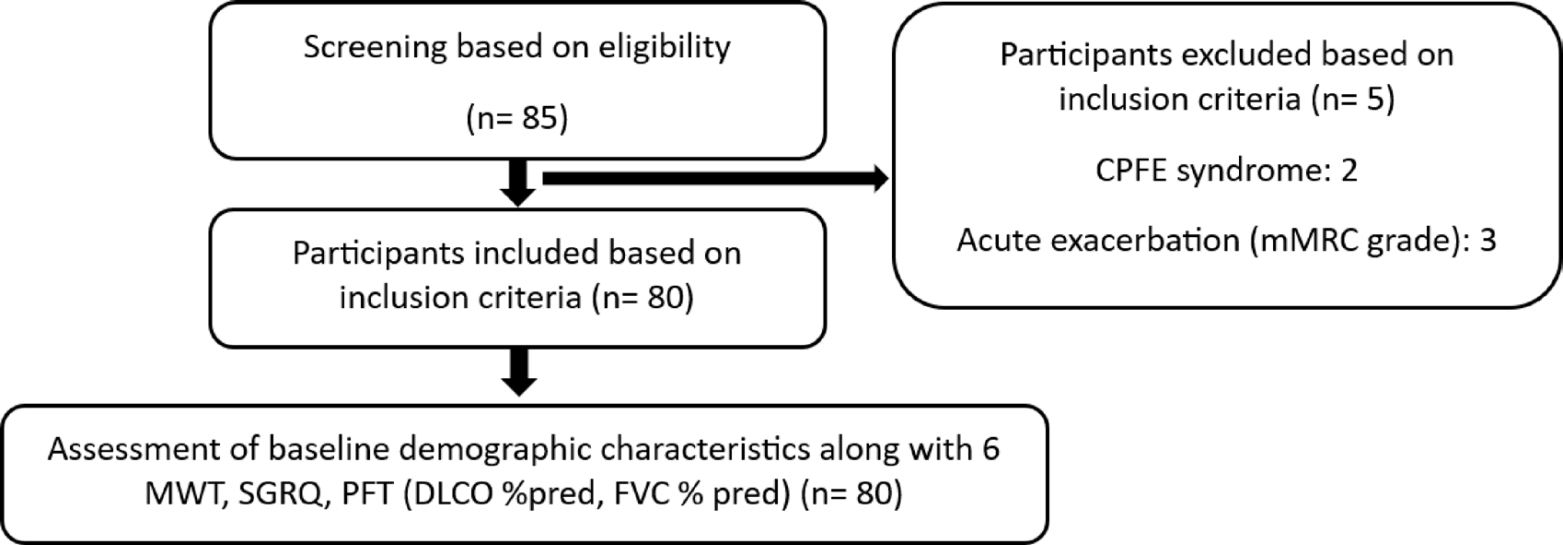
Flow diagram.

Eighty participants with ILD, mean (SD) age 59.5 (13) years, 41 males (51%), were recruited. (Fig. 1) Of the 80 participants, 39 (49%) were diagnosed as Idiopathic Pulmonary Fibrosis (IPF), 19 (24%) as Non-specific Interstitial Pneumonia (NSIP), 9 (11%) as sarcoidosis, 6 (8%) as Rheumatoid Arthritis associated Interstitial Lung Disease (RA-ILD), 7 (9%) as Connective Tissue Diseases associated Interstitial Lung Disease (CTD-ILD). Participant characteristics are shown in Table 1. 

**Table 1:** Demographic characteristics, pulmonary function, exercise capacity and health-related quality of life of participants.

Characteristics	Mean (SD)	Median (IQR)
Age, years	60 (13)	
Age, years		
Males		62 (41,75)
Females		59 (49,74)
Height, cm		163 (146–186)
Height, cm		
Males		162 (149, 175)
Females		165 (146,186)
Weight, kg	50.2 ± 9.5	
Weight, kg		
Males		49 (39, 73)
Females	51.7 (11.11)	
BMI, kg/m^2^	19.1 ± 3.5	
BMI, kg/m^2^		
Males		18 (14, 25.9)
Females		18.3 (14.5, 29.4)
Male: Female, n, (%)	41:39 (51%:49%)	
Diagnosis < 3 years, n, (%)	67 (84%)	
Diagnosis > 3 years, n (%)	13 (16%)	
D_L_CO, % predicted		47 (30–64)
FVC, % predicted		74.5 (41–111)
mMRC scores: n, (%)		
Grade 2	15 (19%)	
Grade 3	65 (81%)	
Previous smoking history, n (%)	26 (33%)	
6MWD, meters	248 ± 46	
SGRQ, Total score		39 (20–54)
ILD subtypes, n (%)		
IPF	39 (49%)	
NSIP	19 (24%)	
Sarcoidosis	9 (11%)	
RA ILD	6 (8%)	
CTD ILD	7 (9%)	
Comorbidities, n (%)		
Diabetes mellitus	16 (20%)	
Hypertension	14 (17.5%)	
Gastro-esophageal reflux	11 (13.7%)	
Pulmonary artery hypertension	11 (13.7%)	

*6MWD* 6 minute walk distance, *CTD ILD* Connective tissue disease associated interstitial lung disease, *DLCO* Diffusing capacity of the lungs for carbon monoxide, *FVC* Forced vital capacity, *ILD* Interstitial lung disease, *IPF* Idiopathic pulmonary fibrosis, *IQR* Interquartile range, *mMRC* modified medical research council, *NSIP* Non-specific interstitial pneumonia, *%* Percentage, *RA ILD* Rheumatoid arthritis associated interstitial lung disease, *SGRQ* St. George’s respiratory questionnaire.

### Diffusion capacity of the lungs for carbon monoxide % predicted (D_L_CO % pred)

The median D_L_CO % predicted values varied based on time since diagnosis, sex, and dyspnea severity. Participants diagnosed with ILD for less than 3 years had a median D_L_CO % predicted of 47 ml/min/mmHg (IQR: 30–64), compared to 44 ml/min/mmHg (IQR: 31–63) for those diagnosed for more than 3 years. Males exhibited a higher median D_L_CO % predicted (49 ml/min/mmHg; IQR: 30–64) than females (45 ml/min/mmHg; IQR: 31–62). Additionally, participants with mMRC Grade 2 dyspnea had a higher mean D_L_CO % predicted (60 ml/min/mmHg; IQR: 53–64) compared to those with Grade 3 dyspnea (44 ml/min/mmHg; IQR: 30–63), indicating a decline in pulmonary function with increasing dyspnea severity. (Table 2).

**Table 2:** Descriptive statistics of participants with for time since diagnosis, sex and dyspnoea scores along with the between group difference.

	Time since diagnosis Mean (SD)			Sex Mean (SD)				mMRC score Mean (SD)			
	Less than equal to 3 years	More than 3 years	Between group difference	Females		Males	Between group difference	Grade 2		Grade 3	Between group difference
D_L_CO %predicted	47(18)	44 (14)	0.5	45 (16.5)		49 (18)	0.07	60 (6)		44 (13)	0.06
FVC %predicted	75 (34.5)	73 (32)	0.4	75 (32.5)		71 (34)	0.06	80 (28)		71 (35)	0.007
6MWD, m	248.7 (47.8)	242.2 (38.2)	0.1	247.7 (57.6)		287.7 (32.6)	0.01	289.3 (20.16)		238.1 (45.23)	0.65
SGRQ, Total score	39 (11.5)	38.4 (10.7)	0.5	37 (15)		40 (13)	0.05	37 (14)		49 (9)	0.008

*6MWD* 6 min walk distance, *D*_L_*CO* Diffusing capacity of the lungs for carbon monoxide, *FVC* Forced vital capacity, *IQR* Interquartile range, *mMRC* modified medical research council, *SGRQ* St. George’s respiratory questionnaire.

### Forced vital capacity % predicted (FVC % pred)

Baseline median (IQR) FVC % predicted for participants with ILD diagnosed for less than 3 years was 75% predicted (41–111) and for those diagnosed for more 3 years was 73% predicted (44–110). The mean difference in FVC % predicted between participants diagnosed with ILD for ≤ 3 years and those diagnosed for > 3 years was 2%, with the associated 95% confidence interval (95% CI) indicating the precision of this estimate. Mean FVC% predicted for females was 75% predicted (41–110, p value = 0.138) and males was 71% predicted (42–111) with no difference between groups (*p* = 0.067). Mean FVC % predicted for participants who reported dyspnoea Grade 2 on mMRC was 80% predicted (42–110) and Grade 3 on mMRC was 71% predicted (41–111). (Table 2).

### 6-minute walk distance (6 MWD)

Baseline mean (SD) 6MWD for participants with ILD diagnosed for less than 3 years was 248.7 m (47.78), and for those diagnosed for more 3 years was 242.2 m (38.20), 6MWD for females was 247.7 m (57.6), and males was 287.7 m (32.6). 6MWD for participants who reported dyspnoea Grade 2 on mMRC was 289.3 m (20.16) and Grade 3 on mMRC was 238.1 m, (45.23). (Table 2)

### St. George’s respiratory questionnaire (SGRQ)

Baseline median (IQR) SGRQ Total score for participants with ILD diagnosed for less than 3 years was 39 (20–53) and for those diagnosed for more than 3 years was 38.4 (20–54) with no difference between groups (*p* = 0.510). Mean (IQR) SGRQ Total score for females was 37 (20–54), and males was 40 (20–53) (*p* = 0.521). Mean (IQR) SGRQ Total score for participants who reported mMRC Grade 2 was 37 (20–53) and mMRC Grade 3 was 49 (41–54) with a significant between-group difference. (Table 2).

**Fig. 2:**
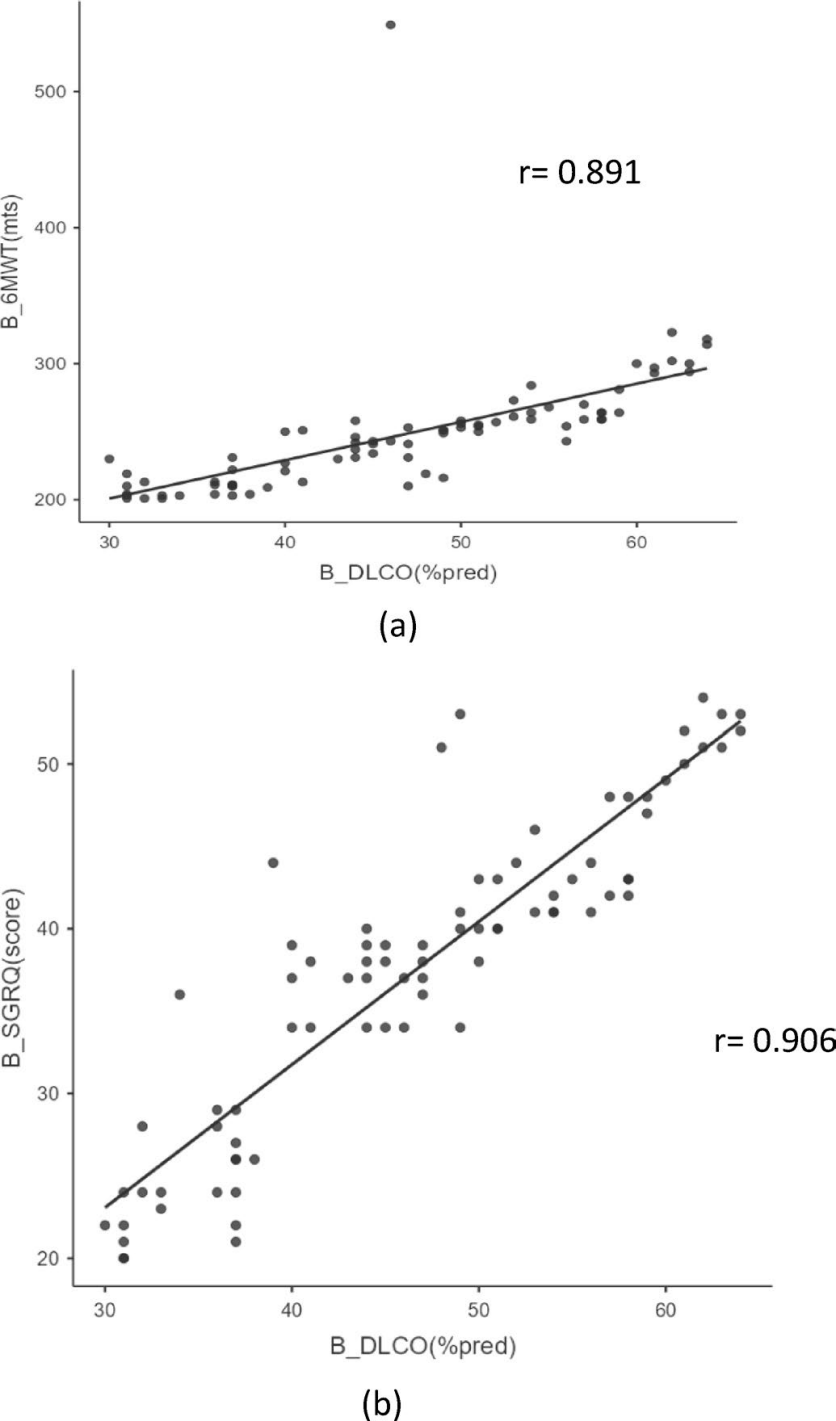
(**a**) Correlation between baseline D_L_CO % predicted with 6 MWD. (**b**) Correlation between baseline DLCO % predicted with SGRQ. *%pred* percentage predicted, *6MWD* 6 Minute Walk Test, *B* Baseline, *DLCO* Diffusing capacity of the Lungs for Carbon monoxide, *mts* meters, *r* Spearman rho value, *SGRQ* St. George respiratory questionnaire.

Table 3 demonstrates the correlations between functional exercise capacity measured by 6MWD and pulmonary function values of D_L_CO% predicted and FVC% predicted for all participants, and for participants with mMRC of Grades 2 and 3. For all participants, there was a strong correlation between 6MWD and D_L_CO% predicted (Spearman rho 0.89, *p* < 0.001). There was no correlation between 6MWD and FVC% predicted (Spearman rho 0.20, *p* = 0.07). There was a strong correlation between SGRQ Total score and D_L_CO% predicted (Spearman rho 0.90, *p* < 0.001). There was no correlation between SGRQ Total score and FVC % predicted (Spearman rho 0.11, *p* = 0.324). (Figure 2a,b)

**Table 3:** For participants with mMRC grades 2 and 3, there were strong correlations between D_L_CO% predicted and 6MWD and SGRQ, but not with FVC % predicted.

	Overall		mMRC Grade 2		mMRC Grade 3	
	6MWD, m (Spearman rho); p-value	SGRQ, Total score (Spearman rho); p-value	6MWD, m (Spearman rho); p-value	SGRQ, Total score (Spearman rho); p-value	6MWD, m (Spearman rho); p-value	SGRQ, Total score (Spearman rho); p-value
D_L_CO % predicted	0.89; <0.001	0.9; <0.001	0.81; <0.001	0.93; <0.001	0.84; <0.001	0.87; <0.001
FVC% predicted	0.20; 0.07	0.11; 0.32	0.14; 0.61	0.05; 0.84	0.14; 0.25	0.03; 0.77

*6MWD* 6 min walk distance, *D*_L_*CO* Diffusing capacity of the lungs for carbon monoxide, *FVC* Forced vital capacity, *SGRQ* St. George’s respiratory questionnaire, *m* metres, *ml/min/mmHg* milliliters per minute per millimeter of hemoglobin, *mMRC* modified medical research council, *%* percent.

### Receiver operating characteristics (ROC) curve analysis

We graded individuals walking less than 300 m of 6MWD as severe impairment, 300–500 m as moderate impairment and more than 500 m as mild impairment. Individuals who score lower than 300 on the 6MWT frequently have a worse prognosis and substantial functional capacity decline. Walking more than 500 m is typically linked to mild functional impairment, whereas the 300–500 m range is regarded as moderate, showing some exercise capacity limitation but not as severe. The cutoff values for categorizing 6-minute walk distance (6MWD) into severe (< 300 m), moderate (300–500 m), and mild (> 500 m) impairment were based on our clinical judgment and assumptions tailored specifically for ILD patients, considering their disease severity and pathology. Given the progressive nature of ILD and its impact on functional capacity, we adopted these cutoffs to reflect the varying degrees of exercise limitation observed in this patient group. These categories align with the general understanding that walking less than 300 m indicates significant functional impairment and a poorer prognosis, while distances above 500 m suggest milder limitations.

We graded D_L_CO levels using commonly recognized thresholds used in clinical practice to assess the severity of gas exchange impairment. D_L_CO values are often expressed as a % of the anticipated value based on age, gender, height, and ethnicity, using the following grading system based on ATS/ERS guidelines^39^:

- Mild impairment: D_L_CO ≥ 60% of predicted.
- Moderate impairment: D_L_CO 40–59% of predicted.
- Severe impairment: D_L_CO < 40% of predicted.

A lower D_L_CO number indicates more severe disease and a worse prognosis. This grading system represents the degree of gas exchange impairment.

**Fig. 3:**
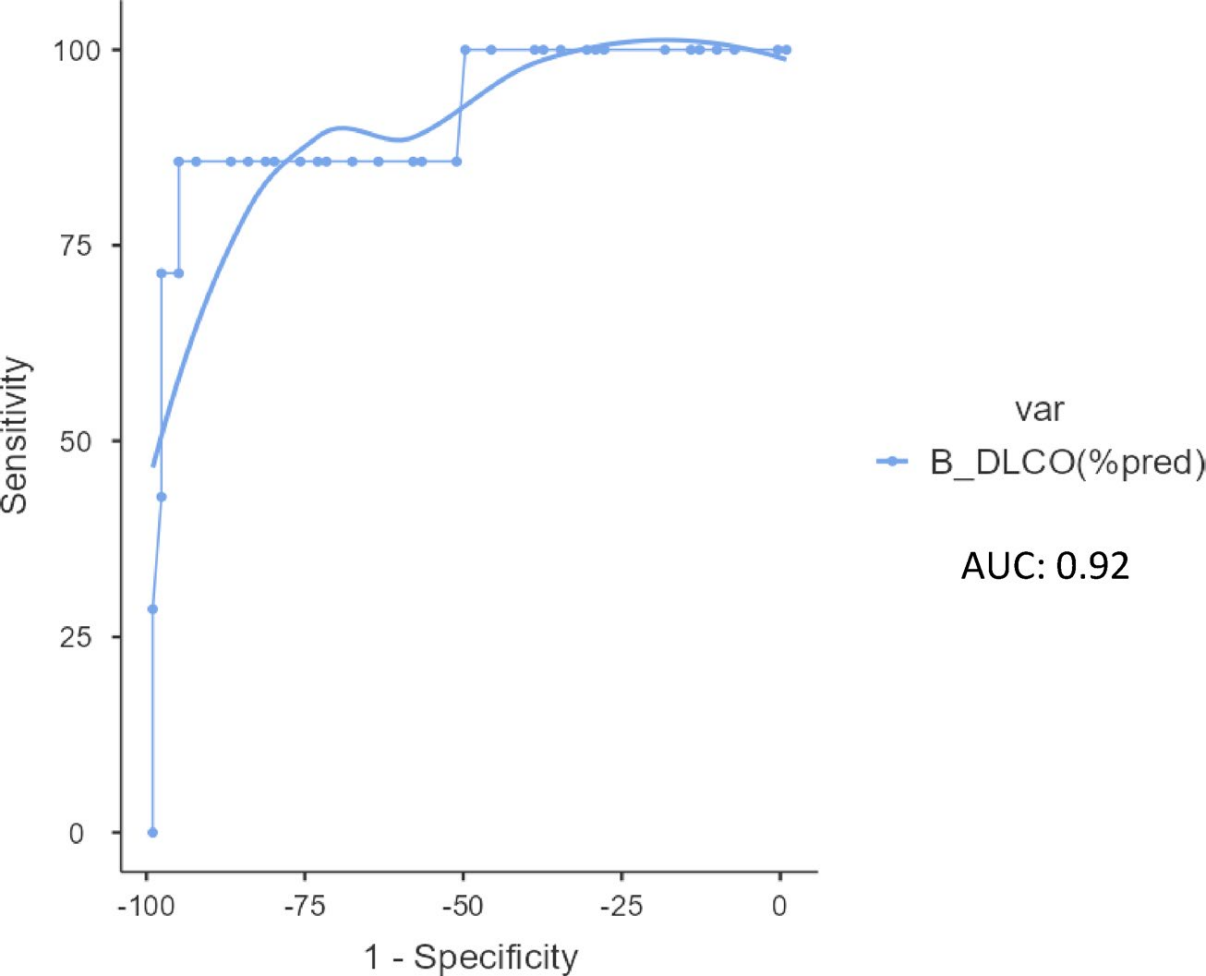
Receiver operating curve (ROC) curve analysis of 6-minute walk test grades across DLCO grades.

The ROC curve analysis shows that the area under the curve (AUC) was 0.92. Our results show that participants walking 300 m to 500 m are likely to have D_L_CO% predicted values between 40 − 60%. Participants walking more than 500 m are likely to have D_L_CO % predicted values between 60% to lower limit of normal. Majority of the participants (58.8%) walking less than 300 m are likely to have D_L_CO% predicted values between 40-60% and 28.7% are likely to have D_L_CO% predicted values less than 40%. (Table 4; Fig. 3).

**Table 4:** Distribution of 6MWD grades across D_L_CO grades with receiver operating curve (ROC) analysis parameters.

6MWD grades	D_L_CO grades		
	Mild, *n* (%) (60%- LLN)	Moderate, *n* (%) (40- 60%)	Severe, *n* (%) (less than 40%)
Mild	0	1 (1.3%)	0
Moderate	6 (7.5%)	0	0
Severe	3 (3.8%)	47 (58.8%)	23 (28.7%)

*6MWD* 6 min walk distance, *D*_L_*CO* Diffusing capacity of the lungs for carbon monoxide, *LLN* Lower limit of normal, *%* percentage.

We graded individuals scoring 0–20 on SGRQ as severe impairment, 21–40 as moderate impairment and more than 41–60 as mild impairment.

**Fig. 4:**
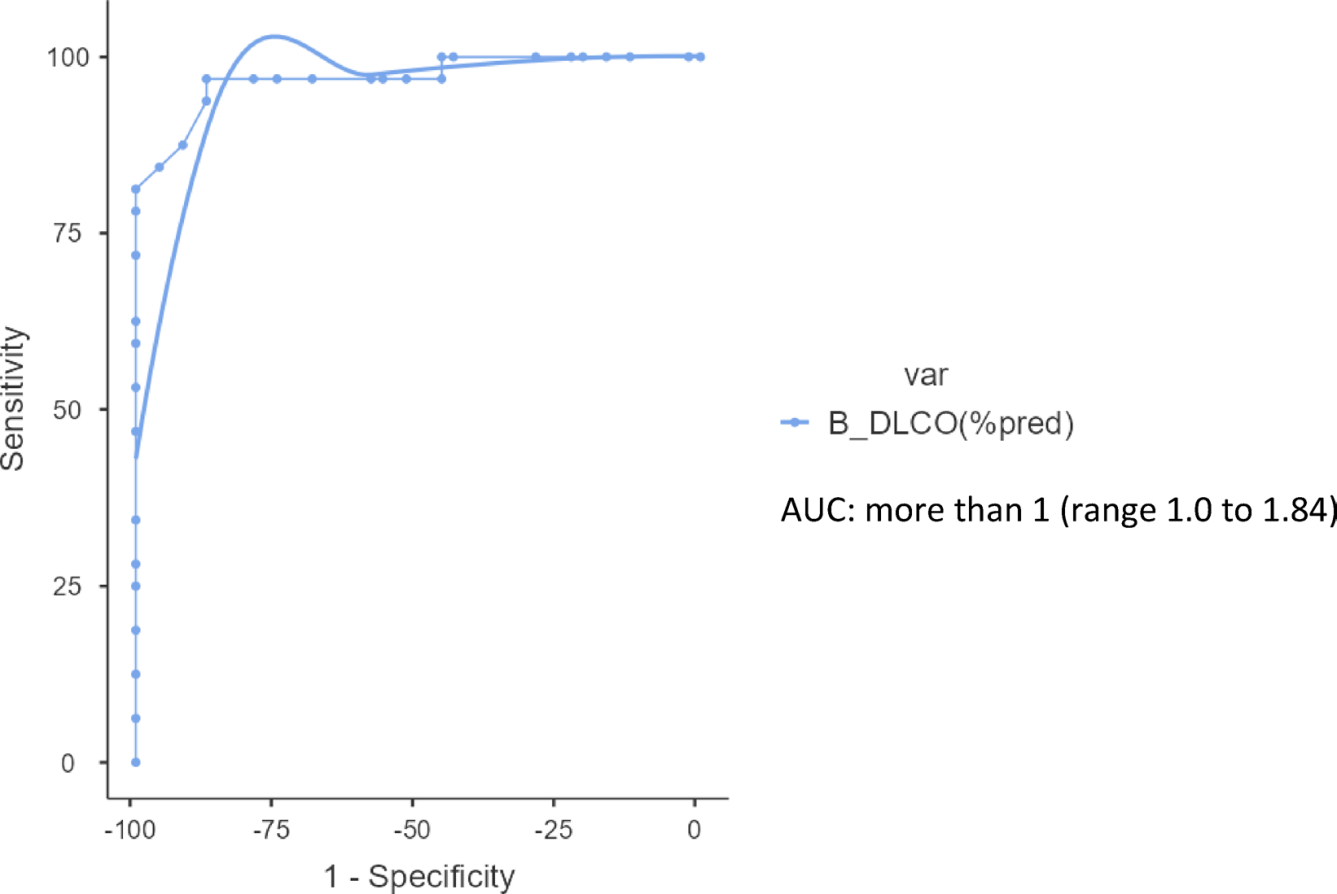
Receiver Operating Curve (ROC) curve analysis of SGRQ grades across D_L_CO grades.

The ROC curve analysis showed that the area under the curve (AUC) for SGRQ grades versus D_L_CO grades was more than 1 (range 1.0 to 1.84). Our results state that SGRQ grade of 41–60 are likely to have D_L_CO values between 40 − 60%. SGRQ grade 21–40 are likely to have D_L_CO values 60% − 40% or less than 40%. SGRQ grade 0–20 are likely to have D_L_CO values from normal to 40%. (Table 5; Fig. 4).

**Table 5:** Distribution of SGRQ grades across D_L_CO grades with receiver operating curve (ROC) analysis parameters.

SGRQ grades	D_L_CO grades		
	Mild, *n* (%) (60%- LLN)	Moderate, *n* (%) (40- 60%)	Severe, *n* (%) (less than 40%)
Severe	9 (11.3%)	48 (60%)	23 (28.7%)
Moderate	0	26 (32.5%)	20 (25%)
Mild	9 (11.3%)	22 (27.5%)	1 (1.3%)

*D*_L_*CO* Diffusing capacity of the lungs for carbon monoxide, *LLN* Lower limit of normal, *%* percentage, *SGRQ* St. George respiratory questionnaire.

## Discussion

This is the first study to investigate the relationships between disease severity, measured by pulmonary function tests (D_L_CO% predicted and FVC% predicted) and dyspnea (using the mMRC scale), and health status parameters such as functional exercise capacity and HRQoL among people with ILD in India. This study found strong correlations between D_L_CO % predicted with both 6 MWD and SGRQ Total score whereas, there were no significant correlations between FVC% predicted with either 6 MWD and SGRQ Total score. Considering time since diagnosis, sex, and reported dyspnea, different patterns in pulmonary function and exercise capacity were identified among participants with ILD according to these characteristics. Individuals diagnosed for less than three years had a decline in D_L_CO % predicted values, but those diagnosed for more than three years had a slightly lower D_L_CO. Males consistently had higher D_L_CO % predicted and lower FVC% predicted compared to females. Dyspnea severity was associated with differences in pulmonary function, with those reporting higher mMRC grades (more dyspnea) often having lower D_L_CO % predicted and FVC% predicted values, suggesting that worsening dyspnea may correlate with greater physiological impairment in ILD. The sex disparity persisted in HRQoL assessments, with a focus on the influence of dyspnea severity on their overall wellbeing.

Survival for people with ILD in India is poor, with up to 46% mortality 4 years after diagnosis, indicating delayed diagnosis due to D_L_CO and high-resolution computed tomography (HRCT) not typically being performed due to access most likely owing to a lack of specialized diagnostic facilities and experience in many rural parts for the given population in India^40–42^ Predictors associated with worse survival include honeycombing of the lungs, current or past history of smoking, and past history of pulmonary tuberculosis^41^ Some of the factors which contribute to the delayed diagnosis are highlighted as lack of standardized care guidelines, lack of defined survival predictors, and high prevalence of pulmonary infections which may also contribute to the high mortality^39^.

Our study showed that a third of participants were past smokers. Tobacco smoking is a significant predictor of poor survival in patients with idiopathic pulmonary fibrosis (IPF)^40,41^ The lower smoking rates in our cohort (particularly among women) reflect regional sociocultural norms, where biomass fuel exposure often supersedes tobacco as an environmental risk factor for ILD. This aligns with findings from a study^40,41^ where biomass-associated ILD demonstrated distinct clinical features. In our cohort, [30%] of non-smokers reported prolonged biomass exposure, suggesting its potential role in disease pathogenesis. Other factors such as genetics, environmental exposures e.g. pollution etc., and the degree of lung fibrosis and honeycombing also play a role in the course of the disease^42^ Inability of patients to perform spirometry, a common test for lung function, is also associated with poor survival, likely because it indicates advanced disease^42^ Due to the expense and accessibility of advanced testing, studies conducted in India indicate that patients frequently place a higher priority on symptom management than on accurate diagnosis. Since symptoms like fatigue and dyspnea predominate their lived experience regardless of quantifiable disease progression by PFTs or HRCT, this could result in a mismatch between perceived disease severity and HRQoL. Reliance on more approachable instruments like the 6MWT or symptom-based evaluations may result from the lack of access to advanced diagnostic tests like D_L_CO and HRCT in many healthcare settings. Given that these alternative measurements may not accurately reflect severity of the disease in comparison to more advanced diagnostics, this could help to explain why the link between disease severity and HRQoL may differ across India.

Regardless of the fact that all patients with ILD experience progressive worsening of dyspnea, which has an influence on their overall quality of life,^43^ few studies have linked health status outcomes to physiological and radiological estimations of disease severity and extent, and none in an Indian population. The study by Martinez et al. (2000) found that chronic dyspnea is a significant contributor to poorer quality of life in patients with ILD, and that lung function variables such as FEV_1_ and FVC do not accurately reflect the impact of the disease on the patient’s health status^9^ with our study demonstrating similar findings. Contrary to other well-cited research, we found no significant relationship between FVC% predicted and HRQoL, which is consistent with Martinez et al. (2000). This disparity could be due to changes in cohort composition (e.g., ILD subtype distribution), the importance of non-spirometric variables (e.g., dyspnea, mental health comorbidities) in triggering HRQoL impairment, or differences in HRQoL evaluation methods. Disease-specific questionnaires may capture respiratory-specific limitations more sensitively than generic instruments, but severe disease stages may dissociate lung function from reported health status due to factors such as oxygen dependency or recurring exacerbations. Our findings demonstrate that HRQoL in ILD is a multicomponent outcome that requires a thorough evaluation that goes beyond spirometry. ILD is often classified into mild, moderate, and severe categories based on FVC% predicted, but this classification system has been criticized for its lack of correlation with patient outcomes^44–50^ In patients with idiopathic pulmonary fibrosis (IPF), a reduction in FVC% predicted of 5–10% following drug treatment is associated with worse survival outcomes, while an improvement change of 2–6% in FVC% predicted is considered the minimum clinically significant difference^16^.

Our study provided a thorough understanding of the relationships between pulmonary function, functional exercise capacity, and quality of life in ILD patients by classifying patients based on their time since diagnosis sex, and levels of dyspnea. Dyspnoea in ILD is likely related to the increased work of breathing due to the interstitial fibrosis which is reflected in low FVC% predicted and reduced D_L_CO^51,52^ Some studies suggest that baseline FVC % predicted can predict the risk of death independently, while others suggest that changes in clinical and physiological variables such as changes in dyspnea score and FVC over 6 and 12 months may provide more accurate prognostic information^52,53^ Customizing treatment techniques requires an understanding of the relationships between various factors such as exercise capacity, D_L_CO, baseline FVC% predicted, changes in dyspnea score, and other variables. As indicated in our study, changes in dyspnea score, D_L_CO, exercise capacity, and health-related quality of life are more than baseline FVC% predicted, enabling physiotherapists and clinicians to prioritize interventions primarily concentrated at relieving symptoms^54^.

The SGRQ is commonly used to assess HRQoL in people with IPF and is used as a key endpoint in some clinical trials^50,16,55^ A previous study found that patients with SGRQ Total scores higher than 30 points had a significantly higher mortality, with a hazard ratio of 2.047^56,57^ In our study the median SGRQ Total score was approximately 39 points, suggesting that our participants were at a high risk of mortality whether they were diagnosed less than or greater than 3 years previously. Previous studies have also demonstrated a correlation between the SGRQ scores and pulmonary function in people with ILD, showing the impact of reduced lung function on HRQoL^58–60^ Our study has added to this literature by demonstrating a strong correlation between functional exercise capacity measured by the 6MWD and D_L_CO, indicating that a decrease in diffusion capacity due to ILD directly impacts functional capacity.

There are some limitations to our study. It was a single-center study, and there may be selection bias. To confirm our findings, further large prospective studies are needed to investigate the relationship between disease severity variables and predictors of health status. Further longitudinal studies should investigate the relationship between these variables and mortality rates. Additionally, our study only included participants with mild to moderate disease severity based on D_L_CO values, and results may differ in more severe disease. The recruitment approach of including 80 participants in the study was intended to produce a representative sample of individuals with diverse disease severity and subtypes.

## Conclusion

This is the first study that investigates the correlations between pulmonary function (D_L_CO % and FVC% predicted), dyspnea severity (mMRC scale), functional exercise capacity (6MWD), and HRQoL (SGRQ Total score) among individuals with ILD in India. D_L_CO % predicted showed strong correlations with 6MWD and SGRQ, while FVC% predicted did not. Our results indicate significant relationships between disease severity (measured by D_L_CO) and both functional exercise capacity (measured by 6MWD) and health-related quality of life (measured by the SGRQ) for participants with Grade 2 and Grade 3 dyspnoea. There were no relationships with disease severity measured by FVC% predicted and a strong positive relationship measured by D_L_CO % predicted. These findings suggest that D_L_CO is a more useful measure than FVC% predicted to determine the impact of ILD on the functional exercise capacity and HRQoL of people in living with this disease in India. Variations in lung function and exercise capacity were observed based on sex, dyspnea severity, and time since diagnosis, with males showing higher D_L_CO % and lower FVC%, and severe dyspnea associated with poorer outcomes. However, where there is limited availability of D_L_CO testing, other tests such as 6-minute walk test and questionnaires such as SGRQ may give an indication of disease severity and the impact of ILD on patients’ lives. These are particularly important findings if access to diagnostic equipment is unavailable.

## Data Availability

All the data collected for the research in question are not published in any other media or data dissemination tool, nor are they stored in specific repositories. However, upon request, the data may be made available at any time. To request access to the data, contact the corresponding author (email address: vaishali.kh@manipal.edu).
